# Family history of arterial hypertension and central adiposity: impact on blood pressure in schoolchildren

**DOI:** 10.1186/s12887-022-03551-4

**Published:** 2022-08-23

**Authors:** Tatiana Aparecida Affornali Tozo, Maria Lourdes Gisi, Caroline Brand, Carla Marisa Maia Moreira, Beatriz Oliveira Pereira, Neiva Leite

**Affiliations:** 1 Postgraduate Program in Education (School of Humanities) – Pontifical Catholic University of Paraná, Curitiba, Paraná Brazil; 2 Postgraduate Program in Health Promotion , University of Santa Cruz do Sul, Rio Grande do Sul Santa Cruz do Sul, Brazil; 3 Research Center in Physical Activity, Health and Leisure (CIAFEL), University of Porto, Porto, Portugal; 4 Institute of Education, University of Minho, Braga, Portugal; 5 Department of Physical Education, Federal University of Paraná, Curitiba, Paraná Brazil

**Keywords:** Risk factors, Waist circumference, Blood pressure, Genetic factors, Schoolchildren

## Abstract

**Background:**

A family history of arterial hypertension is an important risk factor for arterial hypertension. This study aimed to verify the mediating role of high central adiposity in the relationship between family history of arterial hypertension and blood pressure in schoolchildren.

**Methods:**

Cross-sectional study with 118 schoolchildren of both sexes aged between 11 and 17 years. Weight, height, waist circumference and body mass index z score were verified. Somatic maturation was predicted by age for peak growth velocity. The family history of arterial hypertension was verified and defined as hypertensive schoolchildren with systolic blood pressure or diastolic blood pressure. Mediation analysis was used with linear regression models applied by PROCESS macro for SPSS (version 22.0), with significance *p* < 0.05.

**Results:**

It was observed that 34.7% of the students have family history of arterial hypertension, 36% of the girls and 44.2% of the boys have arterial hypertension. In girls, the relationship between waist circumference and systolic blood pressure was direct (β = 0.535 *p* = 0.005), and those with a family history of arterial hypertension and who had a waist circumference greater than those without a family history of arterial hypertension was significant (β = -5,437 *p* = 0.009). Likewise, the relationship between family history of arterial hypertension and systolic blood pressure was attenuated when waist circumference was included in the model (β = -5.544; *p* = 0.103), indicating waist circumference as a mediator with an influence percentage of 19%. For boys, waist circumference is not a mediator of the relationship between family history of arterial hypertension and blood pressure.

**Conclusions:**

Elevated central adiposity was a mediator of the relationship between family history of arterial hypertension and high blood pressure in girls, indicating the importance of family health strategies in the prevention and management of arterial hypertension in children and adolescents.

## Background

Among the non-communicable chronic diseases (cardiovascular, chronic respiratory, cancers and diabetes) arterial hypertension (AH) is a multifactorial clinical disease characterized by persistently high levels of pressure, which causes changes in the function and/or structure of target organs. Risk factors include genetics, ethnic group, age, sex, obesity, sedentary lifestyle [1], high sodium intake, low level of education, and the existence of related comorbidities [2–4]. Therefore, it remains one of the greatest public health challenges worldwide, due to the increased risk of cardiovascular events, including the appearance of AH in the child and adolescent population associated with excess total and central adiposity [5, 6].

The early diagnosis of AH in childhood and adolescence is necessary to recognize this reality and act in the prevention of cardiovascular events, since the change in blood pressure in childhood can predict AH in adulthood [7, 8], simplified cut-off points have even been proposed for screening children and adolescents according to age and sex [9]. As well, family history is important for early screening and for initiating appropriate treatment, as AH is usually asymptomatic [2, 10]. In the child and adolescent population, when both parents are hypertensive, the chance of their children also becoming hypertensive is greater than when only one has AH [11].

For a better approach to this health problem, it is essential to prioritize the most vulnerable population groups and identify as much as possible the risk factors for the development of AH. In addition to genetic predisposition, an environmental aspect that has reached large proportions and is related to AH is physical inactivity and sedentary lifestyle which, associated with inadequate nutrition, result in obesity [12–14], lower cardiorespiratory fitness and metabolic disorders in children and adolescents [15]. The influence of lifestyle on the health of different population groups, including children and adolescents that can lead to numerous diseases, among them, AH is discussed and analyzed in a comprehensive way [16–18].

Therefore, conducting population-based diagnoses of AH associated with family history reinforce the importance of including blood pressure assessment in schoolchildren, together with anthropometric values [19, 20]. In addition, they may be instrumental in establishing predictive risk models, detection, treatment, and control criteria to potentially identify those most and least likely to develop hypertension in adulthood [20, 21].

Thus, observing the main causes of AH, in addition to the potentiating factors of its existence, are important aspects in the attention of public and social policies, which collaborate for the control and prevention of the same [22, 23]. Professional teams, public health centers and all State investments in the fight against AH are a way of seeking to increase the quality of life and combat early deaths. Based on this context, the aim of the present study was to verify the mediating role of excess central adiposity in the relationship between family history of arterial hypertension (FHAH) and blood pressure (BP) in schoolchildren. Therefore, it will be possible to establish the importance of strategies for family health and school environment in the treatment and prevention of AH.

## Methods

### Study population (sample)

This is a cross-sectional study carried out during the period from August to December 2017, consisting of a sample of 118 schoolchildren in the age group from 11 to 17 years (mean age 14.6 ± 1.5 years), 75 girls (mean age 14.9 ± 1.4 years) and 43 boys (mean age 14.1 ± 1.5) from primary and secondary education from two private schools in the city of São José dos Pinhais, Paraná, in southern Brazil. The sample consisted of conglomerates, each associated city school, in which it was considered a conglomerate. The proportional stratified sample procedure was used, so that schools with a larger population contributed more to the sample. Participants were selected by convenience, as the selection was not randomized, but voluntary. In addition, the number of participants was proportional to the number of students from each educational institution. While the sample size calculation was performed to verify the sampling power of the analyzes chosen for this study.

After the consent of the educational institutions, visits were made to the classrooms to invite and present the research objectives to the students and the questionnaire on family history of arterial hypertension was sent to the parents. Data collection was always carried out by the responsible researcher, who provided some health professionals oriented to the evaluations.

The sampling power was calculated posteriorly in the GPower v3.1 program. for the t-test statistic to compare the presence and absence of a family history of hypertension. Effect size (d) 0.50 was assigned, α 0.05. Furthermore, the sample could f hi calculated according eat final sample of this study (118 school). Based on these parameters, the current sampling power of 0.82 for the present study was observed.

Pregnant women, individuals with limitations that prevented them from participating in some procedure, and those who did not have the Free and Informed Consent Terms in accordance with Resolution 466/12 of the National Health Council involving signed human beings were excluded from the study. The present study was approved by the Research Ethics Committee of the Pontifical Catholic University of Paraná, PUC – PR, under registration 2,198,319, and followed the resolution 466/2012 of the National Council of Health in Brazil. The schoolchildren’s parents or legal guardians signed free and informed consent forms.

### Anthropometry

Anthropometric measurements were taken at school in a standardized way, following the procedures recommended by the *Anthropometric Standardization Reference Manual* [24]. Body weight [kg] was evaluated with an Aura model—807—SECA digital scale, with a resolution of up to 100 g and a capacity of 150 kg, with the participant in an orthostatic position, barefoot, feet together in the center of the platform and wearing light clothes. To measure height, a portable stadiometer (AVANUTRI) with millimeter resolution and height of up to 210 cm was used, and the student should have his arms loose along his body and his head positioned in the Frankfurt plane (looking forward), keeping the scapulae and buttocks in contact with the device.

To verify the nutritional status according to the body mass index (BMI), the ratio between body weight (kg) and height squared (m2) was calculated, as well as the body mass index z-score (BMI-z) was calculated according to the *Growth reference data for 5–19 years 17* [25] using the WHO Anthro Plus® *software*, version 1.0.4. Participants with BMI-z between ≥ -2 and <  + 1 were classified as overweight, between ≥ 1 and < 2 as obese, and those with BMI-z ≥  + 2 according to age group and gender were classified as normal weight.

A flexible and inelastic tape measure with a precision of 0.1 cm for the measurement of waist circumference (WC), measured at the midpoint between the last costal arch and the iliac crest. The waist circumference classification followed the cut-off points: WC values ≤ p10: low WC, WC values ≥ p10 and < p75: adequate WC, WC values ≥ 75 and < p90: high WC, and WC values ≥ p90: very high WC. Central obesity was defined as WC > p75 for age and sex [26].

Biological maturation was estimated by the somatic maturation method predicted by age for peak growth velocity (APGV) [27]. This method estimates the difference in maturity (in years) by height and chronological age for boys and girls.

### Blood pressure assessment

BP classification according to age, sex and height percentile following the new Brazilian Guideline on Arterial Hypertension for children and adolescents [2]. BP measurement was performed by nurses in an isolated room in a quiet environment, using automatic pressure devices (OMRON705-IT) [28]. The subject was asked to be seated, calm, rested for at least 5 min of rest, with an empty bladder and without having practiced physical exercises for at least 60 min. The students kept their legs uncrossed, feet flat on the floor, back leaning against the chair with the arm at heart level, supported with the palm facing up and the elbow slightly flexed in which the device was placed on the cuff [29].

Schoolchildren aged up to 13 years with systolic blood pressure (SBP) or diastolic blood pressure (DBP) above the 95th percentile for sex, age and height were defined as hypertensive. In children aged ≥ 13 years as: Normotensive: BP < 120/ < 80 mmHg; High blood pressure: BP 120/ < 80 mmHg to BP 129/ < 80 mmHg; Stage 1 hypertension: BP 130/80 mmHg or up to 139/89 mmHg and Stage 2 hypertension: BP ≥ between 140/90 mmHg [2]. It was obtained as a technical error of measurements (TME) relative to 0% for body mass (BM), 0.14% for height, 0.61% for WC and 0.27% for BP, with evaluators considered experienced because the error was < 1.0%.

The FHAH was assessed through a questionnaire sent to the parents and/or guardians of the students to identify whether the father, biological mother or both were hypertensive. The survey analysis was classified into two categories: “Positive Hypertension Family History and “Negative Hypertension Family History”.

### Statistical analysis

Descriptive data were presented as mean and standard deviation for continuous variables and relative and absolute frequency for categorical variables. The correlation matrix was used for analysis between dependent, independent, and mediating variables. To verify whether the association between FHAH and BP was mediated by WC linear regression models were applied by PROCESS macro for SPSS (version 22.0). The PROCESS macro used bootstrapping methods to test mediation hypotheses [30], using a resampling procedure of 10,000 bootstrap samples.

The objective of this model was to investigate the total (c) and direct (a, b, c’) effects, reflected by the non-standardized regression coefficient and the significance between the independent and dependent variables in each model. The model also explored the indirect effect obtained from the product of the coefficients (a × b), which indicates the change in the dependent variable (SBP and DBP) for each unit change in the independent variable (FHAH) that is mediated by the proposed mediator (WC). The indirect effect was estimated using 95% confidence intervals, considered significant when it does not contain zero.

Thus, the following criteria were used to establish mediation: (I) the independent variable (family history of hypertension) is significantly related to the mediator (WC); (II) the independent variable (FHAH) is significantly related to the dependent variables (SBP and DBP); (III) the mediator (WC) is significantly related to the dependent variables (SBP and DBP); and (IV) the association between independent and dependent variables is attenuated when the mediator is included in the regression model. The analyses were adjusted for the APGV and the probability value *p* ≤ 0.05 was considered significant for all analyses.

## Results

Table 1 presents the main descriptive characteristics of the sample according to sex. The sample consisted of 118 schoolchildren, aged between 11 and 17 years (mean = 14.61, SD = 1.46), with 75 girls (mean = 14.88 SD = 1.37) and 43 boys (mean = 14.14, SD = 1.52). Boys had higher height, WC, and APGV, compared to girls (*p* < 0.05). While the girls had higher levels of DBP (*p* < 0.05).

**Table 1:** Sample characteristics

	**Total**	**Male**	**Female**
	***n* = **118	***n* = 43**	***n* = **75
	**Mean (SD)**	**Mean (SD)**	**Mean (SD)**
Age (decimal)	14.60 (1.46)	14.14 (1.51)	14.87 (1.37)
Body weight (kg)	58.62 (12.73)	59.66 (12.46)	58.03 (12.92)
Height (m)	1.62 (0.08)	1.65 (0.09)	1.61 (0.06)*
BMI (Kg/m^2^)	22.09 (4.32)	21.74 (4.13)	22.29 (4.44)
WC (cm)	69.02 (9.54)	73.32 (9.90)	66.56 (8.46)*
APGV (years)	12.89 (0,82)	13.65 (0.53)	12.46 (0.62)*
SBP (mmHg)	123.62 (13.88)	125.47 (13.76)	122.56 (13.93)
DBP (mmHg)	71.78 (9.74)	69.39 (9.17)	73.14 (9.84)*
	**n (%)**	**n (%)**	**n (%)**
Family history of hypertension			
Positive	41 (34.7)	15 (34.9)	26 (34.7)
Negative	77 (65.3)	28 (65.1)	49 (65.3)

*Caption*: *BMI* Body mass index, *WC* Waist circumference, *APGV* Age peak growth velocity, *SBP* systolic blood pressure, *DBP* Diastolic blood pressure

^*^p < 0.05

**Fig. 1:**
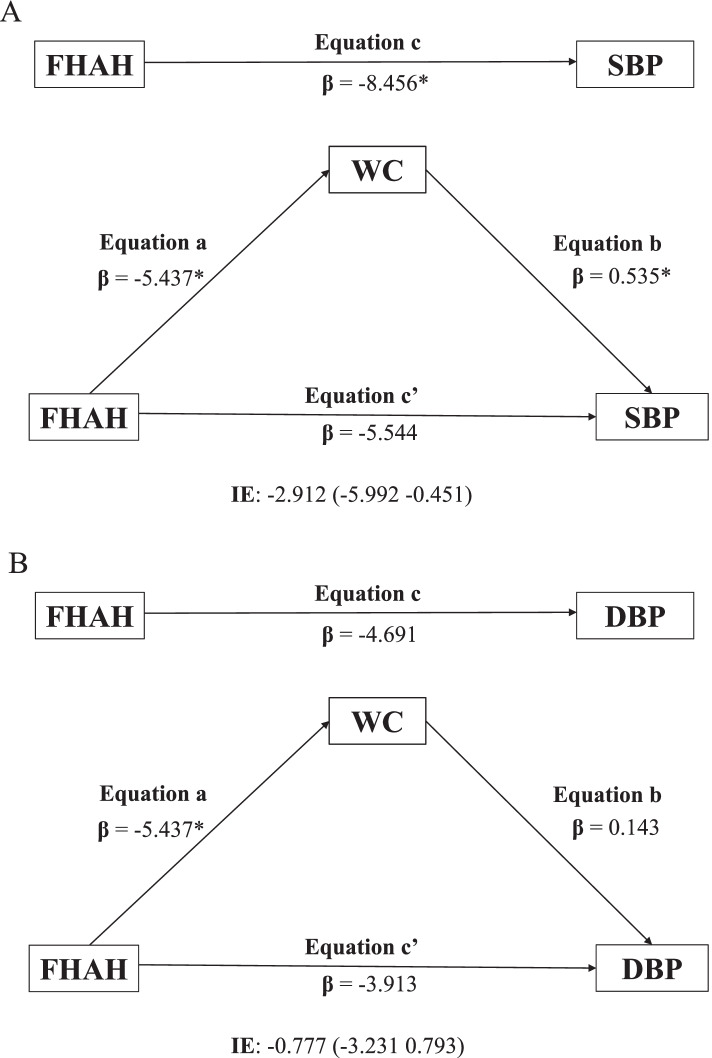
Role of waist circumference as a mediator of the relationship between family history of hypertension and systolic (**A**) and diastolic (**B**) blood pressure in girls. FHHA = Family History of Hypertension; WC = Waist Circumference; SBP = Systolic blood pressure; DBP = Diastolic Blood Pressure; IE = Indirect Effect; *p < 0.05

Figure 1 presents the models used to test the mediating role of WC in the relationship between FHAH and blood pressure in female schoolchildren. Regarding SBP, the first regression equation (a) indicated that girls who did not have FHAH had lower WC, compared to those who had positive FHAH was significant (β = -5,437 *p* = 0.009). In the second equation (c), negative FHAH was inversely associated with SBP (*p* = 0.014). In the third equation (b), the relationship between WC and SBP was positive (β = 0,535 *p* = 0,005). While the fourth equation (c’) indicated that when FHAH and WC were simultaneously included in the model, WC was not associated with SBP (β = -5.544; *p* = 0,103). Thus, the relationship between FHAH and SBP was attenuated when WC was included in the model, indicating that WC was a mediator of this relationship. In addition, it was estimated that the percentage of influence of WC on the relationship between FHAH and SBP was 19%. Regarding DBP, the results indicated that WC does not act as a mediator.

**Fig. 2:**
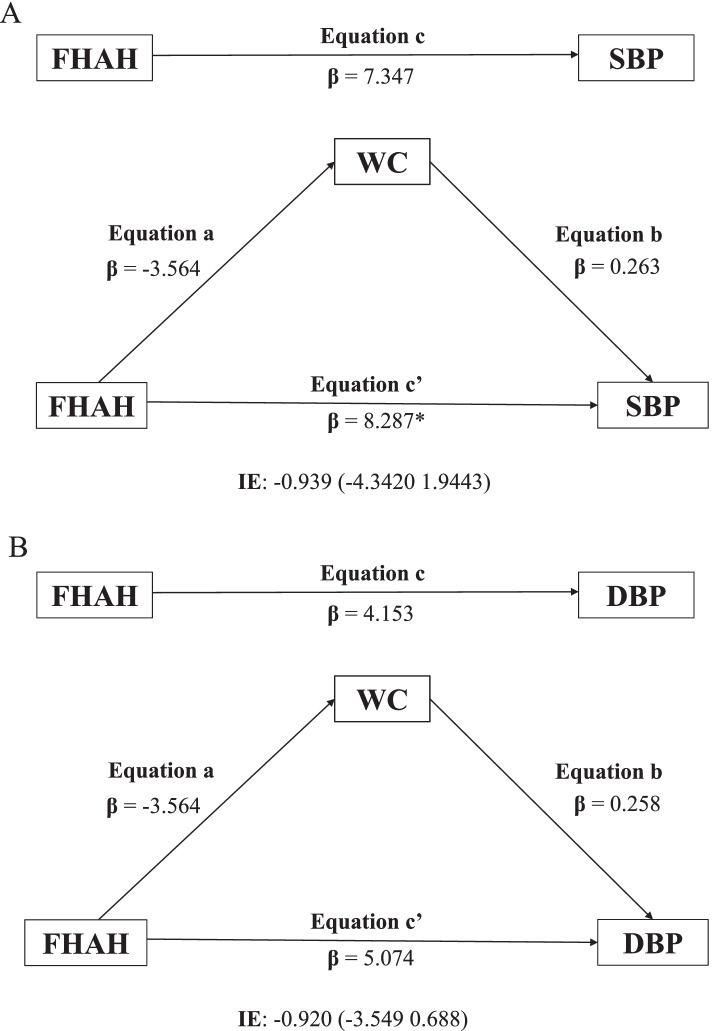
Role of waist circumference as a mediator of the relationship between family history of hypertension and systolic (**A**) and diastolic (**B**) blood pressure in boys; FHAH = Family history of hypertension; WC = Waist circumference; SBP = Systolic blood pressure; DBP = Diastolic blood pressure; IE = Indirect Effect; *p < 0.05

Figure 2 shows the mediating role of WC in the relationship between FHAH and BP in male schoolchildren. The results indicated that WC is not a mediator of the relationship between the FHAH, both with SBP and DBP.

## Discussion

Primary AH is considered a multifactorial disease, but with a strong genetic component, a factor that can influence BP levels and be associated with a higher concentration of inflammatory mediators, which contribute to the development of AH. In this context, the present study sought to identify the mediating role of high central adiposity in the relationship between a family history of arterial hypertension and blood pressure in schoolchildren.

The sample profile revealed that one third of the participants had a positive FHAH. In relation to anthropometric variables, boys presented higher WC compared to girls. Despite this, girls had higher levels of DBP, which suggests that a FHAH may be an important predictive factor for AH [31]. Despite this aspect, other studies have observed a relationship between anthropometric data on obesity and blood pressure elevation [32, 33]. Furthermore, evidence reaffirms the importance of excess weight and abdominal fat as predictors for the presence of hypertension, diabetes, dyslipidemia, and metabolic syndrome, important to identify cardiovascular risks [34, 35].

In the mediation models, WC assumed a more consistent mediating role when verifying the relationship between the family histories of AH and SBP in girls. The percentage of influence of 19% of WC in the relationship between the family history of SBP in females was observed, denoting that central adiposity plays an important role in the high SBP of girls with a FHAH, which implies that they may be in a higher level of risk factor [36]. Corroborating these data, most studies report an important relationship between the presence of high blood pressure and a family history of hypertension, aspects that show the need for investigation to detect the probability of risk of their children developing chronic non-communicable diseases [37, 38].

However, the results indicated that WC was not a mediator of the relationship between the FHAH, with both SBP and DBP among male students. However, most studies have mentioned higher prevalence of AH in this population, which may be related to intra-abdominal fat accumulation, sodium reabsorption, increase in pro-inflammatory cytokines, which causes increased blood pressure [39, 40]. In view of the above, the need for health information is reaffirmed, which includes the identification of genetic and environmental factors, which provide tools that can store, generate, organize and analyze the data necessary to define problems and risks of hypertension, as well as subsidies for management of care policies and referral of schoolchildren to health practices [41]. Adequate treatment requires adequate and regular clinical evaluation of parents and children, which is not common among people with lower income levels, people with low education or residents in remote areas, and people with poor social and health infrastructure [42, 43].

In fact, central obesity, in addition to other extrinsic factors, such as low level of physical activity, hypercaloric diets [44] are among the strongest correlates for predicting future blood pressure elevations [6, 20]. In addition, it is associated with metabolic factors such as excess body weight [45, 46], insulin resistance, higher levels of circulating leptin, and markers of inflammation [47]. On the other hand, irregular consultations, non-adherence to treatment, incorrect medication and few changes in lifestyle and in health-related behaviors of hypertensive patients are also considered the main factors of ineffective control of AH [48]. These factors further increase the risk of complications caused by the disease, which can lead to higher hospitalization rates.

The present work has some limitations that must be considered for the interpretation of the results. Among them, we can highlight the fact that it is a cross-sectional study that does not allow causal associations. In this way, the measurement of SBP and DBP was performed in a single visit to the school, not allowing the assessment in an outpatient setting. Another aspect concerns the parents’ diagnosis of AH having been self-reported using a questionnaire. In view of this, the clinical assessments of blood pressure, as well as anthropometric assessments to analyze the association of these variables, were not performed. Despite the limitations pointed out, the relevance of this study is highlighted in diagnosing high blood pressure levels in schoolchildren associated with their parents’ history of hypertension, as well as associating central obesity as a risk factor for these changes.

The correlates identified in this investigation must take into account all factors to detect schoolchildren and their families at the highest possible risk of AH. The presence of FHAH and anthropometric changes can be used for early prevention and management of this important public health problem, prioritizing periodic assessments and future strategies for more vulnerable population groups with overweight and obesity, low levels of physical activity and with a FHAH.

## Conclusions

The results found in this study provide data on the association of excess abdominal fat and diagnosis of AH in schoolchildren, contributing to important aspects of public health. In addition, WC was identified as a mediator of the relationship between the FHAH and SBP in girls. While in boys there was no significant relationship between the presences of AH and positive FHAH in relation to this variable. Early detection of the relationship between excessive central adiposity and the presence of AH, as well as analyzing these results with socioeconomic and behavioral factors, which can interfere with cardiovascular disease and mortality. We conclude that verifying the relationships between excessive anthropometric measurements and the presence of FHAH are essential in public health actions. Simple and low-cost analyzes that can favor the adoption of policies and control actions to ensure a growth in expectation and a healthier lifestyle in children and adolescents and in adulthood.

## Data Availability

The database used and analyzed in the present study is not publicly available as its information may compromise the participants’ privacy and consent involved in the research. However, the data are available from the corresponding author (EA), upon request.

## Abbreviations

AH: Arterial hypertension
HFAH: Family history of arterial hypertension
WC: Waist circumference
BP: Blood pressure
SBP: Systolic blood pressure
DBP: Diastolic blood pressure
BMI: Body mass index
BMI-z: Body mass index z score
APGV: Age for peak growth velocity
IE: Indirect Effect
